# Green and energy-efficient methods for the production of metallic nanoparticles

**DOI:** 10.3762/bjnano.6.243

**Published:** 2015-12-10

**Authors:** Mitra Naghdi, Mehrdad Taheran, Satinder Kaur Brar, M Verma, R Y Surampalli, J R Valero

**Affiliations:** 1 INRS-ETE, Université du Québec, 490, Rue de la Couronne, Québec G1K 9A9, Canada; 2 CO2 Solutions Inc., 2300, rue Jean-Perrin, Québec, Québec G2C 1T9 Canada; 3 Department of Civil Engineering, University of Nebraska-Lincoln, N104 SEC PO Box 886105, Lincoln, NE 68588-6105, USA

**Keywords:** environmentally friendly methods, Green Chemistry, green reagents, nanoparticles

## Abstract

In the last decade, researchers paid great attention to the concept of “Green Chemistry”, which aims at development of efficient methods for the synthesis of nanoparticles (NPs) in terms of the least possible impact on human life and environment. Generally, several reagents including precursors, reducing agents, stabilizing agents and solvents are used for the production of NPs and in some cases, energy is needed to reach the optimum temperature for reduction. Therefore, to develop a green approach, researchers had the opportunity to investigate eco-friendly reagents and new energy transfer techniques. In order to substitute the harmful reagents with green ones, researchers worked on different types of saccharides, polyols, carboxylic acids, polyoxometalates and extracts of various plants that can play the role of reducers, stabilizers or solvents. Also, there are some reports on using ultraviolet (UV), gamma and microwave irradiation that are capable of reducing and provide uniform heating. According to the literature, it is possible to use green reagents and novel energy transfer techniques for production of NPs. However, these new synthesis routes should be optimized in terms of performance, cost, product quality (shape and size distribution) and scale-up capability. This paper presents a review on most of the employed green reagents and new energy transfer techniques for the production of metallic NPs.

## Introduction

Nanoscience and nanotechnology are defined in several ways. According to the strictest definition, nanotechnology is the production or use of materials and structures so that at least one of their dimensions is in the range of 1–100 nm [1–3]. The properties of nanostructured materials differ remarkably from those of bulk materials due to variation in specific characteristics, such as size, morphology and distribution [4–5]. They exhibit a higher surface to volume ratio that consequently increases their surface energy and biological effectiveness [6–7]. Therefore, nanotechnology attracted the attentions of many researchers in different research areas, such as physics, chemistry, biology, and engineering [8]. Their investigations resulted in development of materials with new structures, such as nanoparticles (NPs), nanolayers (NLs) and nanotubes (NTs) that have greatly influenced all aspects of human life [9–11]. Currently, a vast number of nanostructured materials with different properties are produced at the lab-scale that may be implemented in different applications. It is highly predictable that NPs with proven applicability will be taken forward to large-scale production [12].

Among different nanostructured materials, metal NPs have a variety of potential applications in versatile areas, such as electronics, chemistry, energy, and medicine [13]. There are many methods for production of NPs, such as lithography, laser ablation, aerosol techniques radiolysis, and photochemical reduction. Generally, these methods are costly, energy intensive or they can be harmful to human and environment [14–15]. For example, the production of nanomaterials through chemical methods involve the use of dispersants, surfactants or chelating agents to prevent the agglomeration of particles. Most of these reagents can be considered environmental pollutants, if they are going to be used in large scale production [16]. As a consequence, there have been growing concerns about the environmental issues of large-scale production of nanomaterials. Therefore, environmentally friendly procedures should be developed that lead to a reduction of cost, energy, product loss and the emission of pollutants [6,8,17–18]. However, the production of monodispersed nanomaterials by using cheap and non-toxic reagents remains a challenge for researchers and more studies are needed to achieve high quality products with sustainable commercial viability [6,13–14,19–20]. Recently, biological systems including microbes and fungi as reactors and plant extracts as precursors have been intensively explored [14]. In another approach, green reagents, such as saccharides, polyols and proteins, and new energy transfer techniques can substitute harmful reagents and conventional heating methods in a typical chemical reaction. In this review, the recent investigations in the past decade on green reagents and energy transfer techniques for the production of metallic NPs are reviewed.

## Review

### Applications of nanotechnology

Due to smaller size and large specific area, NPs exhibit exceptional properties for applications in different fields including chemistry (catalysis, sensors, and polymers), physics (optics and electronics), biotechnology (detection and control of micro-organism), and medicine (drug development and immunoassays) [4,21–23]. For example, NPs made from platinum, palladium, gold, silver, and copper have applications in biological labeling, optoelectronics, photography, photonics, surface-enhanced Raman scattering (SERS) detection and catalysis of chemical reactions. Furthermore, biocompatible and functionalized NPs have applications in diagnosis and treatment of cancer. For these two purposes, fluorescent and magnetic nanocrystals for detection of tumors and also nanosystems for delivery of anticancer drugs have been demonstrated [24–34]. In Table 1, the application of different metallic NPs is summarized.

**Table 1:** Applications of nanotechnology in different fields.

application	NP material	references

technology		

optics (optical and electro-optical devices, spectrally selective coatings)	Au Ag ZnO Pt	[5,25,35–44] [5,25,37,41–43,45] [46] [37]

medicine		

diagnosis and treatment (monitoring of cancer, development of new drugs (anticancer), drug delivery, fabrication of implants, healthcare product (glucose sensor, antimicrobial agent))	Ag Au Pd CuO Pt CuO Fe_3_O_4_ ZnO carbon	[5–7,9–10,13,22,25,37,41–43,45,47–73] [14,20,25,35,37,39,56,70–71,74–88] [51] [89] [37] [89] [90–92] [46] [93]
DNA study (labeling, detection, sequencing)	ZnO Au Ag	[46] [74] [53]
decontamination from organics		

water purification	ZnO Au	[46] [14]
site remediation (soil, air)	Fe Fe-Pd TiO_2_	[94–95] [96] [97]

industry		

chemical reaction (electrocatalysts, photocatalysts, pigments)	Ag Au Pt Pd Au–Ag Pd–Ag Au–Pd ZnO Pt–Pd	[5,9,19,25,37,42,45,58,60,73,98–102] [14,25,35,37,74,77,79–80,98,101,103] [37] [16] [104] [105] [106] [46] [107]
energy systems (heat transfer devices, energy storage (electrical batteries), solar energy absorption))	Ag Au Au–Ag CuO	[5,42–43,108] [108] [108] [89]
electronics (microelectronics, nanoelectronics, high-conductivity elements fabrication, optoelectronics)	ZnO Au Ag Pt	[46] [14,35–36,39,77,80,109] [9,44,60,100,102,109] [109]

analytical and measuring instruments		

surface-enhanced Raman spectroscopy (SERS)	Ag Au	[41,44,53,56–57,73,99–100,110–112] [14,35,41,56]
sensors	Ag Au CuO ZnO	[5,43] [35,39,82,113] [89] [46]

biology		

biological study (biological labeling, targeted biological interactions , detection of reporter molecules, diagnostic biological probes, biosensing , fluorescent probes)	Ag Fe_3_O_4_ Au	[5,42–43,54,66] [90] [36,114]

consumer products		

household items (detergents, soaps, shampoos, cosmetic products, and toothpaste)	Ag Au Pt Pd	[7,43,52,115] [115] [115] [115]
food	Ag	[7,43]

### Green Chemistry metrics

Green Chemistry is gradually integrated into new scientific and industrial developments to be aligned with the global demand to reduce the emission of toxic waste into environment. These sustainable processes should consider 12 major principles of Green Chemistry before putting them into practical effect. These principles are set to minimize the use of toxic reagents and maximize the yield of products [34,116].

**Inhibition of waste generation:** Prevention of the generation of wastes is preferred to their purification. In this sense, the formation of any unusable by-products or the loss of consumed energy can be taken into account as waste. Each form of waste has its own impacts on the environment depending on its nature, toxicity, quantity, or the way it is released [117–118]. Different strategies, such as controlling the morphology can be taken into consideration to prevent the generation of undesirable products during NPs fabrication.

**Atom economy:** Atom economy addresses the maximization of product yield in terms of raw materials consumption, so that the maximum number of atoms of the precursors is found in the product. The ideal reaction would contain all the atoms of raw materials [119–120]. Employing fewer number of reactants through selection of reagents capable of playing multiple roles (e.g., polysaccharides as reducing and capping agents) for production of metallic NPs is a common strategy that increases the atom economy of reactions [121].

**Less harmful chemical processes:** Synthesis procedures should be designed to be capable of consuming and producing materials that have little or no toxicity to the environment and human health [11]. Using biologically produced compounds, such as coffee and tea extract for reduction of Ag and Pd precursors to NPs is reported as an example of green methods with non-hazardous reactants [51].

**Designing safer materials:** Gathering information about the properties and impacts of molecules on the environment and their transport and fate in the biosphere is necessary to achieve sustainability. By understanding their properties, scientists can design safer molecules for the environment and humans [122–123]. For example, one of the problems with NMs is the impurities that they carry and which could have toxic effects on the environment. To prevent such a problem, using modern purification strategies can be useful whereby the impurities can be retained [18].

**Less toxic solvents and auxiliaries:** In Green Chemistry, solvents are considered a bigger challenge since they are used in larger amounts than the other materials [124–125]. In addition, most of the conventional solvents have problems, such as toxicity, flammability, and corrosion. Their solubility and volatility may result in contamination of air, water and soil and also can increase the risk of exposure to workers. The recovery of these solvents through conventional distillation process is often energy-intensive. Therefore, in case of NPs synthesis, scientists focused on safer solutions, such as solventless systems or non-toxic solvents, i.e., the water/glycerol system [92,126–127].

**Energy efficiency:** Reducing the activation energy of the chemical processes by selecting appropriate precursors in a way that the conversion can take place at ambient temperature is an important target to reduce energy consumption [128]. Enhancing the energy efficiency of a chemical process and using alternative energies, such as solar and wind power are considered to be important components of the solution [118]. Incorporation of starch as a reducing agent for synthesis of Ag–Au bimetallic NPs at room temperature is a good example of an energy-efficient process since there is no need to increase the temperature of the reaction medium [104].

**Renewable feedstock:** Increasing the share of renewable sources either for raw material and energy are very important. The largest renewable source for energy is biomass [129]. There are also many examples for using renewable material in synthesis of NPs including cellulose, chitin, starch and glycerol [130–133].

**Reduction of derivatives:** Derivatization processes, such as blocking, protection, and temporary physical or chemical alteration should be limited, since they introduce additional chemicals and increase energy consumption and waste generation [116,118]. In the synthesis of metallic NPs, using biopolymers such as chitosan can eliminate the need to use capping agents [56,86,99].

**Catalysis:** Selecting proper catalytic reactions can enhance the overall efficiency of the process by decreasing the activation energy and increasing product selectivity. These advantages can result in less energy and raw material consumption, and also less waste generation [118]. For example, polyoxometalates (POMs) can act as a photocatalysts in the synthesis of metallic NPs so that the reactions can take place at room temperature within several minutes [134].

**Degradability:** Chemical products should not be long-standing in the environment and therefore chemists should design them so that at the end of their life span, they can easily cleave into simpler and non-toxic molecules [135]. For example, using edible and biodegradable polymers, such as gum ghatti for stabilizing NPs ensures a short life span of the product after release into the environment [66].

**Real-time analysis of pollutants:** The monitoring of the concentrations of different chemicals is crucial for the prevention of undesirable events. This approach can save energy and prevent accidents and also unwanted production of by-products that may need further degradation steps. Conventional analytical methods involve pretreatment steps that generate waste and, therefore, green analytical chemistry can be defined as the use of measurements that generate less waste and are thus safer to the environment and to human health [136–137]. In the field of nanotechnology, the real-time monitoring of size and shapes of nanostructures is of high importance even though it is very challenging. There are reports on developing innovative systems, such as grazing-incidence small-angle X-ray scattering setup that showed a high sensitivity to control the required parameters of the production of NPs [138].

**Inherently safer chemistry:** All types of required substances for a chemical process should be selected so that the all hazards and risks of the system, such as toxicity, flammability and explosivity are minimized to prevent accidents [116]. In recent years, researchers tried to get rid of toxic and flammable reagents, such as hydrazine, sodium borohydride, carbon monoxide, and dimethyl formamide (DMF) in the synthesis of NPs [90].

### Green synthesis of NPs

Metal NPs can be produced and stabilized by various physical and chemical approaches. Among them, the reduction of a precursor and the capping of the produced NPs with various stabilization agents is of interest because of the robustness and feasibility of these processes. The properties of NPs including size, shape and stability strongly depend on the reaction conditions, interaction of precursors with reducing agents, and adsorption of stabilizers at NPs. Therefore, researchers worked on different precursors, solvents, reducing agents, stabilizers and also reaction conditions to control the properties of NPs. However, the synthesis processes can exert serious problems on the environment. In most of the recent reported synthesis processes, organic solvents such as dimethylformamide (DMF) and toxic reducing agents, such as sodium borohydride are heavily employed. Most of these solvents and reagents can exhibit potential risks to environment and organisms [34,87].

In the last 10 years, the awareness about the environmental issues of chemical processes has increased and led scientists to focus on Green Chemistry for synthesis of nanostructured materials [32,51]. Using safer reagents, less harmful solvents and renewable feedstock and energy are among the major issues that deserve attention in a Green Chemistry approach [4,87]. For green synthesis of NPs, three major principles of Green Chemistry should be considered including the selection of (I) green solvents, (II) non-toxic reducing agents, and (III) harmless stabilizers [21,26,32,51,112,139].

Biochemical, biological and biomimetic processes are attracting the attention of researchers due to their viability and potential in minimization of waste [62,92]. For example, synthesis of NPs in bio-directed systems and using bio-molecules as templates for production of inorganic molecules has attracted biologists and chemists [81].

Synthesis and stabilization of NPs from bio-compatible materials is of high importance for their applications in medical diagnosis and therapeutics [87]. Among the vast number of available natural raw materials, polysaccharides and biologically active products extracted from plants provide largest feedstock for this process [78]. The hydroxyl and other functional groups in polysaccharides can play a major role in the reduction and stabilization steps during the production of metallic NPs. Also, phytochemical compounds have biological activities and can be considered as a renewable resource for the synthesis of metallic NPs [62].

Natural polymers form the other major category of organic materials that are used for the stabilization of metallic NPs. For this purpose, the repeating unit of the polymer should have functional groups to bind the metal atoms. The size of metallic NPs can be logically controlled by using polymers as soft support [21].

Using microwave irradiation can reduce energy requirement and provides a more environmentally friendly approach in comparison to conventional methods. Furthermore, microwave irradiation provides uniform nucleation and growth conditions for nanomaterials, since it offers rapid and uniform heating of the constituents [112].

### Green reagents

#### Saccharides

Potara et al. found that chitosan (CTS) is not only capable of reducing and stabilizing, but it can aksi act as a scaffold for the formation of Au NPs. Their results indicated that the formation, size, shape and crystalline structure of Au NPs in a polymeric matrix are strongly influenced by the reaction temperature. At *T* = 100 °C and *T* = 20–50 °C, Au NPs had sizes of 18 and 27 nm, respectively, while at lower temperatures (4–10 °C), they observed anisotropic nanosheets of different shapes within the range of 40–200 nm [86]. Also, Wei et al. used CTS in aqueous solution of AgNO_3_ and HAuCl_4_ to act as reducing agent and scaffold for the formation of Au and Ag NPs. They used surface plasmon resonance (SPR) analysis to confirm the formation of NPs [29]. In related reports, they used TEM analysis and observed that their Ag NPs have spherical shape with diameters of 6–8 nm [56,99]. An et al. prepared Ag NPs using CTS as stabilizer agent after stirring the aqueous solution for 30 min at 30 °C. Their SEM micrographs showed a regular spherical shape with less than 20 nm in size and also their TGA analysis exhibited higher thermal stability of Ag–CTS in comparison to CTS. Their microbial experiments showed that the antibacterial performance of Ag–CTS is more than either Ag NPs or CTS [72]. Sun et al. prepared Au NPs using HAuCl_4_ as a precursor and CTS as the reducing agent and stabilizer in a 30 h reaction at 55 °C. According to TEM images, the sizes of Au NPs were 10–50 nm. During the synthesis, they observed a decreasing trend in intrinsic viscosity [η] of chitosan that implied degradation of chitosan chains due to the reaction with HAuCl_4_ [140]. Also, several researchers worked on derivatives of CTS. For example, Wang et al. produced biocompatible chitosan–ninhydrin (CHIT-NH) bio-conjugates for the use as reducing agent of Au precursors at 37 °C. They claimed that this new reducing agent can overcome the non-uniform spatial distribution of stabilizers to form organized one-dimensional assemblies of Au NPs with average diameter of about 18 nm [114]. Long et al. used oligo-chitosan [(GlcN)*_x_*] as stabilizer to prepare biocompatible Ag NPs from AgNO_3_ at room temperature. Their NPs were stable in a pH range of 1.8–9.0 and their average sizes were between 5 and 15 nm. They found that Ag NPs can be stable in NaCl solution. However, they are aggregated in the presence of NaNO_3_ or NaH_2_PO_4_ [141]. Laudenslager et al. used CTS and carboxymethyl chitosan (CMC) as stabilizing agent for production of Pt, Au and Ag NPs. These two biopolymers gave similar size distributions, while CMC showed higher aggregation due to lower availability of amines and the reduced cross-linking ability. The average sizes of Pt, Au, and Ag NPs were about 3.5, 23, and 7.5 nm respectively. According to their FTIR data, the amine and amide functionalities had the most interaction in CTS, while in CMC, the alcohol functionalities played this role [142]. Although CTS is a green reagent, using NaBH_4_ as reducing agent indicates that the process is not totally green. Huang and Yang utilized CTS and heparin as reducing and stabilizing agents at 55 °C in synthesis of Au and Ag NPs, respectively. Their results suggested that amino groups in chitosan and sulfonic groups in heparin can provide enough electrostatic attractive force for the formation and stabilization of the Au and Ag NPs. They observed an increasing trend in the size of the Ag NPs while increasing the concentration of Ag^+^ or heparin. The particle sizes of CTS stabilized Au NPs and heparin stabilized Ag NPs were in the range of 7–20 nm and 9–29 nm, respectively [143].

**Figure 1:**
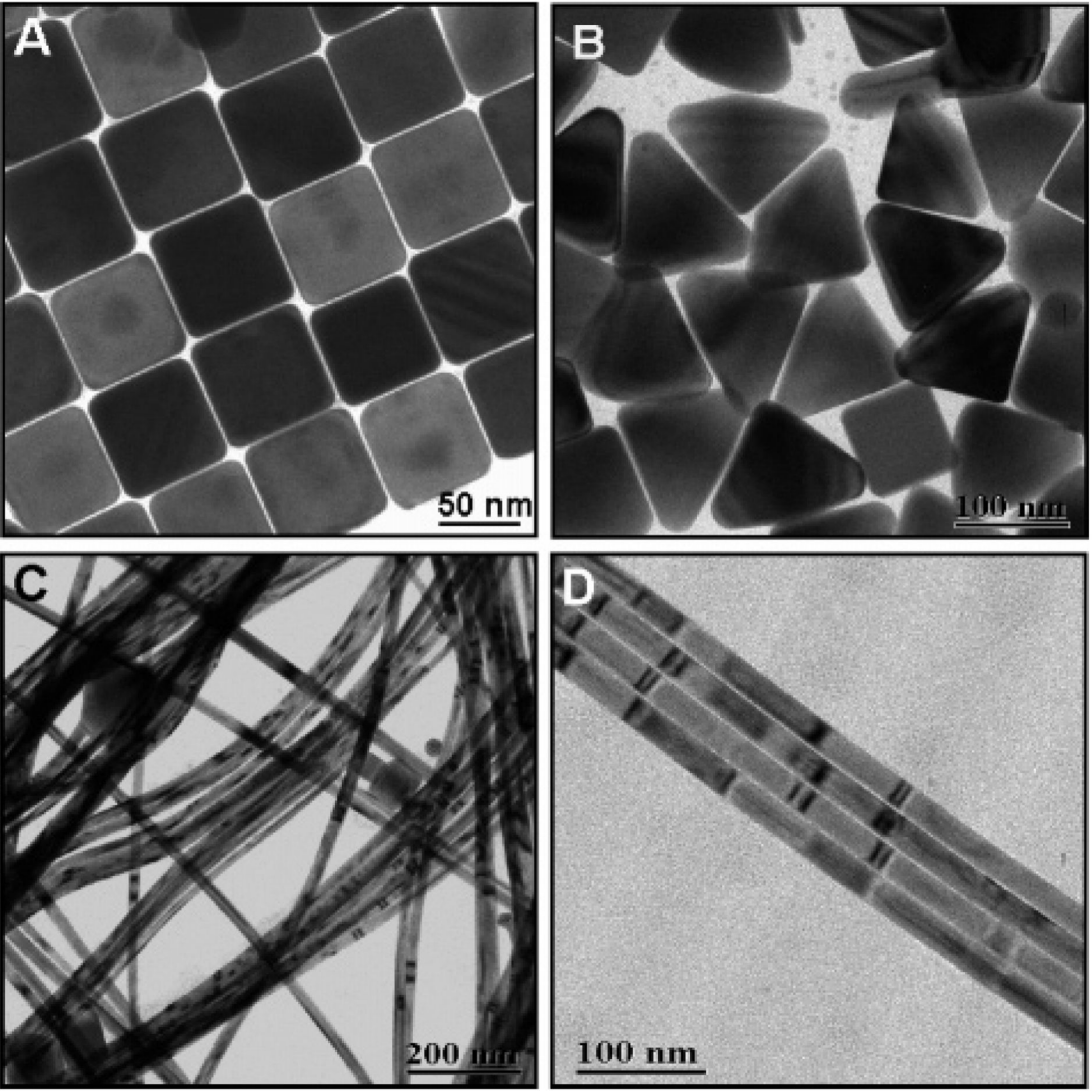
TEM images of Ag NPs: (a) cubes; (b) triangles; (c) wires; (d) an alignment of wires. Reproduced with permission from [145]; Copyright (2005) American Chemical Society.

**Figure 2:**
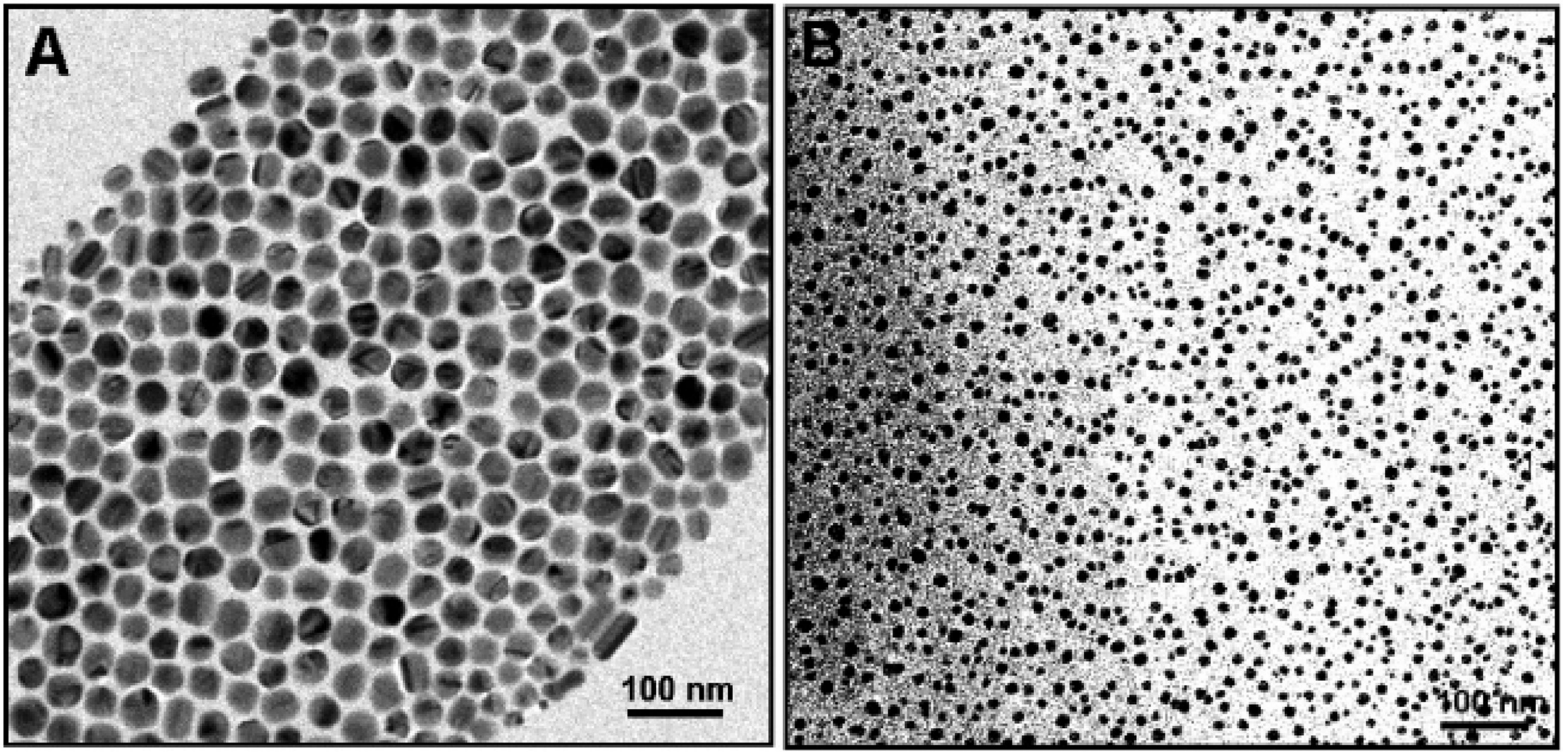
TEM images of Ag colloids synthesized at 120 °C for 8 h. Reproduced with permission from [145]; Copyright (2005) American Chemical Society.

Raveendran et al. reported a method for the synthesis of Au, Ag, and Au-Ag NPs in aqueous media, using glucose as the reductant and starch as stabilizer. The prepared bimetallic NPs were uniform and their sizes were within the quantum size domain (less than10 nm), where their electronic properties are size-dependent. They observed no signs of aggregation even after several months of storage [26,108]. He et al. reduced [Ag(NH_3_)_2_]^+^ ions by glucose in aqueous solution and then they added Al(NO_3_)_3_ into solution to synthesize Ag nanosheets. They claimed that the in situ generated Al(OH)_3_ influenced the formation of Ag nanosheets. The produced nanosheets in 60 min reaction had a thickness of 20–30 nm [111]. Sun and Li produced colloidal carbon micro and nanospheres from glucose in a hydrothermal process (at 160–180 °C for 4–20 h) and used this functionalized carbon for in situ encapsulation of Ag and Au NPs. The size of the produced NPs with this method could be controlled in the range of 8–50 nm [144]. In a similar work, Yu and Yam used D-glucose in a hydrothermal process for synthesis of Ag NPs. As depicted in Figure 1 and Figure 2, they achieved interesting assembles of particles, such as cubes, triangles, wires and spheres [145]. Soukupova et al. reduced the complex cation of [Ag(NH_3_)_2_]^+^ by D-glucose to achieve Ag NPs in a single-step process. They studied the influence of different surfactants, i.e., cationic (cetyltrimethylammonium chloride, CTAC), anionic (sodium dodecylsuphate, SDS) and non-ionic (Tween 80) at 20 °C on fundamental characteristics of Ag NPs. They found that in comparison to unmodified NPs, non-ionic surfactants can improve the polydispersity from 8.5 to 2.5%, and ionic surfactants can reduce the zeta potential of Ag NPs from −20 to −50 mV, which is favorable for stabilization. They concluded that non-ionic surfactants can form a layer with inhibition function to prevent the formation of other nuclei and consequently lead to monodisperse NPs [100]. Lu et al. prepared super-paramagnetic Fe_3_O_4_ NPs utilizing gluconic acid as stabilizing agent and α-D-glucose as reducing agent at mild temperatures (60 and 80 °C) in aqueous media. They obtained spherical NPs with comparable size (≈12.5 nm) and polydispersity to conventional methods [90]. Darroudi et al. produced Ag NPs with gelatin and glucose as reducing and stabilizing agent for Ag^+^ ions in aqueous media. They investigated the effect of temperature (28, 40 and 60 °C) on particle size and found that the size of NPs decreases with increasing temperature. They also observed that using gelatin solutions resulted in smaller particle sizes compared to gelatin–glucose solutions, due to the rate of the reduction reaction. Their instrumental analysis including XRD, UV–vis spectrometry, TEM, and AFM confirmed the formation of NPs with a quite narrow particle size distribution. The size of their NPs was less than 15 nm [21]. Kvitek et al. compared the performances of four different sugars including xylose, glucose, fructose and maltose in reduction of AgNO_3_ in the presence of ammonia and production of spherical Ag NPs in a single-step reduction process at 20 °C. They found that decreasing the ammonia content from 0.2 to 0.005 M can decrease the particle size from 380 down to 45 nm. For higher concentrations of ammonia (0.2 M) there are slight differences in the particle sizes of Ag NPs produced by the four sugars (352–380 nm). But at low ammonia concentrations (0.005 M), the average size of particles in the case of fructose (161 nm) are three times more than that in the case of other sugars (47–57 nm) [57]. In a similar study, they used galactose and lactose as reducing agents and achieved Ag NPs with the average particle size of 50 and 35 nm at 0.005 M ammonia concentrations [65]. In another work, they produced spherical Ag NPs with an average diameter of 26 nm, and polydispersity of 2.3%. They also investigated the capability of different ionic and non-ionic surfactants and also polyethylene glycol (PEG) and polyvinylpyrrolidone (PVP) in surface modification and stabilization of Ag NPs produced by reaction of AgNO_3_ and D-maltose. According to their results, sodium dodecyl sulfate (SDS), polyoxyethylenesorbitan monooleate (Tween 80) and PVP (*M*_w_ = 360000) were superior stabilizers for aggregation of Ag NPs [64].

Tai et al. used starch and glucose to reduce AgNO_3_ to Ag NPs in a spinning disk reactor (SDR). Their reaction at room temperature took place in 10 min and the sizes of their NPs were less than 10 nm. They observed that high a AgNO_3_/starch ratio or high glucose concentration can increase the yield up to 70%. They also found that the selection of pH and dispersing agent are highly influential on the NP sizes [146]. Deka et al. prepared starch–Au composite NPs by ultra-sonication for 20 min at 25 °C and used α-amylase for the enzymatic release of Au NPs. Their TEM analysis showed well-dispersed spherical NPs with 10–30 nm diameter [83]. Vigneshwaran et al. utilized soluble starch as reduction and stabilization agent in the synthesis of Ag NPs at 121 °C and 15 psi for 5 min. The sizes of prepared NPs were in the range of 10–34 nm. They observed no aggregation in aqueous solution over three months at ambient temperature. They confirmed the entrapment of Ag NPs inside the helical chains of amylose by iodometric titration method [87]. Li et al. produced Cd–Se bimetallic NPs using sodium selenosulfate (Na_2_SeSO_3_) as precursor and soluble starch as stabilizer at ambient temperature and pressure within 2 h. Their NPs were of the cubic structure with the average particle size of 3 nm according to XRD analysis and evaluation with the Scherrer equation [147]. Xia et al. used renewable degraded pueraria starch (DPS) as reducing and capping agent for the synthesis of Au–Ag bimetallic NPs at room temperature within ca. 24 h. They claimed that most of the synthesized particles had uniform spherical morphology with average diameter of 32 nm [104].

Kemp et al. synthesized Au and Ag NPs using 2,6-diaminopyridinyl heparin (DAPHP) and hyaluronan (HA) as both reducing and stabilizing agents and HAuCl_4_ and AgNO_3_ as precursors at 70 °C. Both reducing agents resulted in high stability under physiological conditions, though the particles size distribution for heparin was narrower (7 nm for Ag and 10 nm for Au) than that of hyaluronan (5–30 nm for both Au and Ag NPs) since diaminopyridine group in heparin formed stronger bonds with NPs. According to their study, Au– and Ag–heparin NPs show considerable anticoagulant and inflammatory properties which is promising for various applications [70]. In a similar report, they used DAPHP and HA for production of Ag NPs from AgNO_3_ and studied their antimicrobial properties. According to this study, Ag–HA and Ag–DAPHP are more stable at physiological salt concentrations than metallic NPs and they show remarkable antimicrobial activity [55]. In another study, they found that Ag– and Au–DAPHP have potential applications in treatment of angiogenesis accelerated disorders, such as cancer and inflammatory diseases [71].

Cai et al. used the nanoporous structure of cellulose hydrogels to synthesize and stabilize Ag, Au, and Pt NPs through hydrothermal process. They found that by increasing AgNO_3_ concentration, the particles size increases gradually from 8 to 11.4 nm at 80 °C and 24 h. Also, reaction time and temperature had direct influence on particle size. The average sizes of Ag, Au and Pt NPs, calculated by Scherrer equation, were 12.3, 6.5, and 4.4 nm respectively. The particle sizes, obtained by TEM analysis, were in good agreement with Scherrer equation [37]. Chen et al. employed carboxymethyl cellulose sodium (CMS) both as reducing and stabilizing agent for production of Ag NPs from AgNO_3_. They employed microwave with the heating power of 0.4 kW to enhance the hydrolysis of CMS in the absence of catalyst in aqueous solution and used CMS hydrolyzate to reduce Ag ion. They found that decreasing AgNO_3_ and increasing CMS concentration (0.04% for 0.1 mM AgNO_3_) will lead to smaller NPs. According to their results, the concentration of CMS has very small effect on distribution of particle size, while an increase in the concentration of AgNO_3_ results in broader size distribution. The NPs produced in this method had an average size of about 15 nm [17].

Jang et al. used dextran (dex), a readily available polysaccharide, both as reducing and stabilizing agents to synthesize dex–Au NPs from HAuCl_4_. The stability of Au NPs is enhanced due to cross-linking of aminated dextran chains on the surface of NPs using epichlorohydrin (C_3_H_5_ClO). The average diameters of their NPs were 80 nm [82]. Morrow et al. used diethylaminoethyldextran as reducing and stabilizing agents to produce Au NPs from Au^3+^ solution at 50 °C for 7.5 h. They found that the performance of dextran is strongly dependent on pH so that in alkaline solutions, the Au^3+^ ions are rapidly reduced to spherical NPs and their sizes range from 18 to 40 nm depending on pH, temperature, and the Au^3+^/dextran ratio. However, in acidic conditions, the reduction is very slow and large Au NPs with different shapes are formed [35].

Saha et al. utilized calcium alginate gel beads as a template for Ag and Au NPs through a green photochemical process using UV light source (365 nm wavelength) for 40 min. In this process, alginate can serve as both reducing and stabilizing agent. The particles had spherical morphology and their sizes were less than 10 nm for both Ag and Au. Their sorption experiment showed that the more Au atoms than Ag atoms are loaded on calcium alginate [98].

Venkatpurwar and Pokharkar mentioned a single step method for synthesis of Ag NPs by using sulfated polysaccharide extracted from marine red algae (*Porphyra vietnamensis*) in a 15 min reaction at 70 °C. The produced NPs showed SPR centered at 404 nm with average particle size measured to fall within the range of 13 nm. Their FTIR study admitted the role of sulfate groups of polysaccharide in reduction of AgNO_3_. Also, the zeta potential measurement (−35.05 mV) confirmed the capping of anionic polysaccharide on the surface of NPs which is responsible for the electrostatic repulsion and consequently stability at wide range of pH (2–10) and electrolyte concentration (up to 10^−2^ M of NaCl) [63].

Thekkae Padil and Cernik used gum karaya (GK) to produce copper oxide (CuO) NPs from CuCl_2_ at 75 °C for 60 min. According to their FTIR results, different sugars, amino acids and fatty acids are responsible for the stabilization processes. They also observed that by changing the concentration of precursor, one can obtain NPs with average particle diameter from 7.8 to 4.8 nm [89].

#### Polyols

Shameli et al. used polyethylene glycol (PEG) and β-D-glucose as stabilizing and reducing agents, respectively, to produce colloidal Ag NPs from AgNO_3_ at 60 °C. They studied the properties of Ag NPs at different reaction times and found that the average particle sizes were 10.60, 11.23, 15.30 and 25.31 nm for different mixing times of 3, 6, 24 and 48 h, respectively. According to the measured zeta potential of 54.5 mV, they concluded that the synthesized Ag NPs had acceptable stability [4]. In another study, they studied the antibacterial activity of different sizes of Ag NPs against two different bacteria and observed that Ag NPs with smaller size have a higher antibacterial activity [62]. Li et al. synthesized Ag NPs using PEG-200 as reducing and stabilizing agent and AgNO_3_ as precursor at ambient temperature within 6 h. Their analysis showed that the Ag NPs are spherical and stable for several weeks and the particle sizes are below 5 nm. PEG can also act as environmentally-friendly solvent and its hydroxyl groups can form complexes with metallic ions and consequently reduce them to NPs [110]. Likewise, Yan et al. used PEG-400 to produce Ag NPs at room temperature from AgNO_3_ in 10 h. Relatively narrow size distributions were apparent for the products. Similarly, the NPs were in the size range from 8 to 10 nm [148]. In another study, Roy and Lahiri synthesized radioactive ^198^Au NPs using PEG-4000 as reducing agent without any other organic solvent. Their particle sizes ranged from 15 to 20 nm [149]. Chin et al. used PEG as the solvent and stabilizer for producing Fe_3_O_4_ NPs by thermal decomposition of iron acetylacetonate (Fe(acac)_3_), which is a non-toxic precursor. They found that by changing reaction time and concentrations of precursor and surfactants, one can control the shape and size of Fe_3_O_4_ NPs. According to them, the average size of Fe_3_O_4_ NPs increases from 2 to 7 nm when the concentration of precursor increases from 0.1 mmol to 8 mmol [91].

Zhang et al. used tannic acid (TA), a water-soluble polyphenol, as the reducing agent to prepare Ag NPs supported on graphene (Ag NPs–GN) in a single-step process over 90 min. They reacted AgNO_3_ and graphene oxide (GO) with TA simultaneously and observed that GO sheets were impregnated with many Ag NPs with diameters up to 20 nm [73].

Kasthuri et al. synthesized anisotropic Au and quasi-spherical Ag NPs using apiin to reduce AgNO_3_ and HAuCl_4_ at room temperature within 60 s. Apiin, an extracted compound from parsley and celery, has eight OH groups and can act also as stabilizing agent. They observed that the size and morphology of the synthesized NPs can be controlled by changing the precursor/apiin ratio. According to their TEM micrographs, the average sizes of the Au and Ag NPs were 21 and 39 nm respectively [38].

#### Carboxylic acids

Lai et al. produced superparamagnetic Fe_3_O_4_ NPs from FeCl_3_ using a mixture of water and glycerol as solvent and L-arginine as stabilizing agent. L-arginine is an amino acid that is naturally produced and therefore it is considered to be a green reagent. The average size of the synthesized Fe_3_O_4_ NPs is reported to be 13 nm [92]. Although they employed green reagents for the production of NPs, using an autoclave at 200 °C for 6 h increased the energy requirement of the whole process. In another study, Hu et al. reduced Ag^+^ to Ag NPs using L-lysine or L-arginine, and stabilized it with soluble starch. In comparison to Lai et al. they reduced the energy requirement using microwave irradiation for 10 s at 150 °C. According to the TEM micrographs, the average particle size of the produced Ag NPs was 26.3 nm. They found that increasing the microwave power from 30 to 120 W can reduce the heating time and particle size from 23 to 28 nm [112].

Kora et al. synthesized Ag NPs from AgNO_3_ in an autoclave at 120 °C and 15 psi. In their reaction, gum kondagogu (*Cochlospermum gossypium*), a natural biopolymer with several hydroxyl and carboxylate groups, was used as a reducing and stabilizing agent. They studied the influence of gum particle size, gum concentration, AgNO_3_ concentration and reaction time on the synthesis of Ag NPs and found that by increasing the concentrations of gum and AgNO_3_, the efficiency of NP production is enhanced. Likewise, by increasing the autoclaving time, more hydroxyl groups are converted to carbonyl groups which in turn increase the reduction of Ag ions. The average size of the synthesized spherical NPs was around 3 nm [54]. In another study, they used gum ghatti (*Anogeissus latifolia*) as a reducing and stabilizing agent for the synthesis of spherical Ag NPs from AgNO_3_. They observed that by increasing reaction time, the efficiency of NP synthesis increases and it is attributed to the higher reduction capacity of the gum. They concluded that hydroxyl and carboxylate groups of the gum help the complexation of Ag ions during process [66].

Kumar et al. used amino acid based phenolic compounds as reducer and stabilizer for production of Ag NPs from AgNO_3_ at room temperature. They stated that amino acids have reactive hydroxyl groups and their structural variations can result in the production of spherical and prism-like NPs [22].

#### Polyoxometalates

Polyoxometalates (POMs) are anionic structures with transition metal atoms in their highest oxidation state. These materials can exhibit tremendous structural variety and interesting properties such as reversible electron exchange behavior that make them ideal candidates for homogeneous-phase electron transfer processes [150–151]. POMs can be used in synthesis of metallic NPs, since their solubility in water and capability for participating in multi-electron redox reactions without structural changes [152].

Zhang et al. studied the capability of the mixed-valence polyoxometalate β-H_3_[H_4_P(Mo^V^)_4_(Mo^VI^)_8_O_40_]^3−^ (POM) both as a reducer and a stabilizer at room temperature. They found that the morphology of the Au NPs can be modified by manipulating the initial concentrations of the POM and HAuCl_4_. For *C*^0^_POM_ = 0.5 mM and [metallic salt]/[POM] = 1, the size of NPs was below 10 nm and it decreased with reducing *C*^0^_POM_ [151]. Zhang et al. used K_9_[H_4_PV^IV^W_17_O_62_] (HPV^IV^) clusters as the reducer and stabilizer for production of Pd NPs from K_2_PdCl_4_ in acidic aqueous solutions. They also admitted that the starting molar ratio of precursor to POM has influence on formation of Pd NPs and reported different size (15–50 nm) for NPs in different precursor to POM ratios [150]. Also, Troupis et al. used K_4_[SiW_12_O_40_] as reducer, photo-catalyst, and stabilizer for production of Au, Ag, Pt and Pd NPs in aqueous solution at pH 5. They used a 1000 W Xenon arc lamp as illumination source to trigger the reaction. The Au and Ag particles were spherical with a diameter of 13.1 and 15.3 nm, respectively. However, Pd and Pt NPs had unclear morphology with the size of 5.0 nm and 2.7 nm [134]. Keita et al. used oxothiometalate, Na_2_[Mo_3_(µ_3_-S)(µ-S)_3_(Hnta)_3_], as reducer and stabilizer for production of Au NPs in aqueous medium at room temperature. The majority of their particles ranged from 9 to 10 nm. They also found that the ratio of Au precursor to POM governs the dispersion of shapes and sizes so that by increasing this ratio from 2 to 4, the size of particle increase from 5 to 54 nm [36]. In a related report, they used mixed valence POMs (Mo^V^–Mo^VI^) including H_7_[β-P(Mo^V^)_4_(Mo^VI^)_8_O_40_] (**1**), (NH_4_)_10_[(Mo^V^)_4_–(Mo^VI^)_2_O_14_(O_3_PCH_2_PO_3_)_2_ (H_2_OPCH_2_PO_3_)_2_]·15H_2_O (**2**), and [P(Mo^V^)_8_(Mo^VI^)_4_ O_36_(OH)_4_(La(H_2_O)_2.5_Cl_1.25_)_4_]·27H_2_O (**3**), to produce Pt and Pd NPs from K_2_PtCl_4_, K_2_PdCl_4_, and PdSO_4_ as precursors in aqueous media at room temperature. The stabilization capability of these mixtures followed the order of **1** > **2** >> **3**. In the case of POM **1** and POM **2** the precursor to POM ratio did not affect the size of NPs but for POM **3** the average size of the NPs increases from 1.7–2 nm to 2.5–4 nm by increasing the precursor to POM ratio from **1** to **2** [153]. They also used α_2_-H_4_PV^V^W_17_ (POMs) to reduce [PdCl_4_]^2−^ to Pd NPs and reported a narrow distribution around 3 nm for NPs [154]. Dolbecq et al. employed two POMs, namely (NH_4_)_18_[(Mo^V^_2_O_4_)_6_(OH)_6_(O_3_PCH_2_PO_3_)_6_]·33H_2_O and [(Mo^V^_2_O_4_)_3_(O_3_PCH_2_PO_3_)_3_(CH_3_AsO_3_)]·19H_2_O for synthesis of Pt and Pd NPs from K_2_PtCl_4_ and K_2_PdCl_4_. Similarly, they observed that the nature of POMs and the precursor to POM ratio can influence the size of NPs [155].

#### Alcohols

Chen et al. studied the fabrication of Pt–Pd bimetallic NPs using ethanol, as reducing agent under mild reaction conditions, and graphene nano-sheets (GNs), as supporting material. As it was expected, changing the molar ratio of the starting precursors, determine the shape of NPs on GNs. They also tried carbon black as support for NPs. According to their calculations, the particle sizes were 7.9 nm for Pt–Pd NPs supported on GNs, 10.2 nm for Pt–Pd NPs on carbon black, 17.3 nm for Pd NPs on GNs and 20.4 nm for flower-like Pt NPs supported on GNs [107]. Safaepour et al. studied the capability of geraniol for reduction of AgNO_3_ to Ag NPs in aqueous solution of PEG-4000 using a microwave oven (with power of 850 W) for 40 s. The sizes of produced NPs ranged from 1 to 10 nm with an average size of 6 nm [47].

#### Other reagents

**Figure 3:**
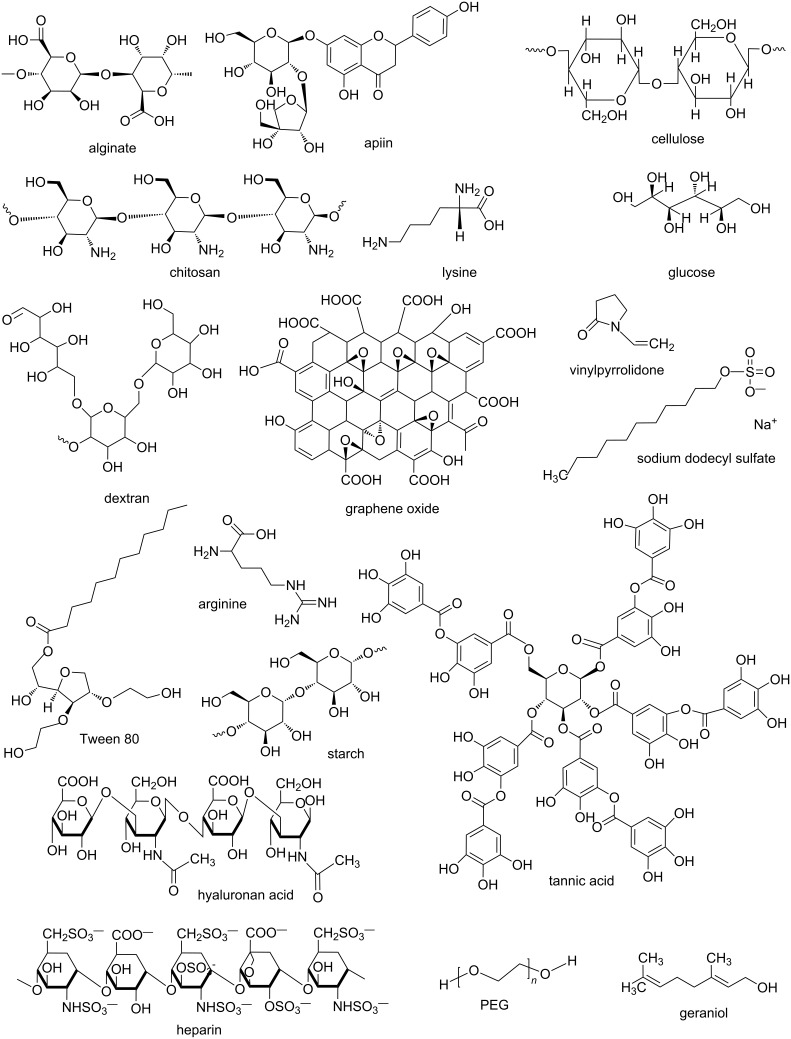
Molecular structures of different green reagents used for synthesis of NPs.

Guidelli et al. studied the production of Ag NPs from AgNO_3_ solution using natural rubber latex (NRL) extracted from *Hevea brasiliensis* at 100 °C for 60 min. Their spherical NPs ranged from 2 to 100 nm. According to their results, lower AgNO_3_ concentration led to formation of smaller particles and higher AgNO_3_ concentration can lead to formation of aggregates. Using FTIR spectra, they found that the ammonia which is used for conservation of the NRL, participate in the reduction of Ag ions and also the *cis*-isoprene moieties help stabilization of NPs [8]. Li et al. produced bimetallic Pd–Ag NPs from AgNO_3_, K_2_PdCl_4_ using graphene oxide (GO) nanosheets as reducing agent, support and stabilizer. The synthesis process took place at 84 °C for 3 h for reduction of metallic ions and 200 °C for 24 h for reduction of GO. The bimetallic NPs were smaller than 10 nm [105]. Different green reagents that researchers tested for synthesis of NPs are listed in Table 2. The molecular structures of different green reagents are shown in Figure 3.

**Table 2:** Summary of synthesized NPs with different green reagents.

NP material	precursor	reducing agent	stabilizer	support	size (nm)	ref.

Au	HAuCl_4_	chitosan	chitosan	—	10–50	[140]
Ag	AgNO_3_	NaBH_4_	chitosan	—	<20	[72]
Au	HAuCl_4_	chitosan	chitosan	—	18–200	[86]
Ag and Au	AgNO_3_ and HAuCl_4_	chitosan	–	chitosan	ND	[56]
Ag	AgNO_3_	chitosan	–	chitosan	6–8	[99]
Au	HAuCl_4_	CHIT–NH^a^	–	CHIT–NH^1^	18	[114]
Ag, Au and Pt	AgNO_3_, AuCl_3_ and H_2_PtCl_6_	NaBH_4_	CMC^b^	—	3.5 (Pt), 23 (Au), and 7.5 (Ag)	[142]
Au	HAuCl_4_	chitosan	chitosan	—	7–20	[143]
Ag	AgNO_3_	—	(GlcN)*_x_*^c^	—	5–15	[141]
Ag	AgNO_3_	heparin	heparin	—	9–29	[143]
Au, Ag and Au–Ag	AgNO_3_ and HAuCl_4_	glucose	starch	—	<10	[26,108]
Ag	[Ag(NH_3_)_2_]^+^	glucose	—	—	20–30	[111]
Ag	[Ag(NH_3_)_2_]^+^	D-glucose	SDS^d^, Tween 80^e^ or CTAC^f^	—	50 (SDS), 65 (Tween 80) and 66 (CTAC)	[100]
Fe_3_O_4_	FeCl_3_·6H_2_O	α-D-glucose	gluconic acid	—	12.5	[90]
Ag	AgNO_3_	gelatin	gelatin	—	<15	[21]
Ag	[Ag(NH_3_)_2_]^+^	four sugars^g^	—	—	45–380	[57]
Ag	[Ag(NH_3_)_2_]^+^	D-maltose	SDS^d^, Tween 80^e^ or PVP 360^h^	—	26	[64]
Ag	AgNO_3_	glucose	starch	—	10	[146]
Au	HAuCl_4_	H_2_O_2_	starch	—	10–30	[83]
Ag	AgNO_3_	starch	starch	—	10–34	[87]
CdSe	CdCl_2_·2.5H_2_O, Se powder and Na_2_SO_3_·7H_2_O	—	starch	—	3	[147]
Au–Ag	AgNO_3_ and HAuCl_4_	DPS^i^	DPS^i^	—	32	[104]
Ag and Au	AgNO_3_ and HAuCl_4_	HA^j^	HA^j^	—	5–30 for both	[70]
Ag	AgNO_3_	DAPHP^k^	DAPHP^k^	—	11	[55]
Ag and Au	AgNO_3_ and HAuCl_4_	DAPHP^l^	DAPHP^l^	—	10 (Au) and 7 (Ag)	[70]
Ag and Au	AgNO_3_ and HAuCl_4_	DAPHP^k^	DAPHP^k^	—	14 (Au) and 10–30 (Ag)	[71]
Ag, Au and Pt	AgNO_3_, HAuCl_4_·3H_2_O and PtCl_4_	—	cellulose	cellulose	11.4 (Ag), 7 (Au) and 5.6 (Pt)	[37]
Ag	AgNO_3_	CMS^l^	CMS^l^	—	15	[17]
Au	HAuCl_4_·3H_2_O	dextran	dextran	—	80	[82]
Au	HAuCl_4_	DEAE–dextran^m^	DEAE–dextran^m^	—	18–40	[35]
Ag and Au	AgNO_3_ and HAuCl_4_	CA^n^	CA^n^	—	<10 for both	[98]
Ag	AgNO_3_	SP^o^	SP^o^	—	13	[63]
Ag	AgNO_3_	β-D-glucose	PEG^p^	—	10.6–25.31	[4]
Ag	AgNO_3_	sugar	PEG^p^	—	11.23	[62]
Ag	AgNO_3_	PEG^p^	PEG^p^	—	<5	[110]
Ag	AgNO_3_	PEG^p^	PEG^p^	—	8–10	[148]
^198^Au	H^198^AuCl_4_	PEG^p^	PEG^p^	—	15–20	[149]
Fe_3_O_4_	Fe(acac)_3_^q^	PEG^p^	PEG^p^	—	2–7	[91]
Ag/GN	AgNO_3_	TA^r^	—	GN^s^	20	[73]
Ag and Au	AgNO_3_ and HAuCl_4_	apiin	apiin	—	21 (Au) and 39 (Ag)	[38]
Fe_3_O_4_	FeCl_3_	—	L-arginine	—	13	[92]
Ag	AgNO_3_	L-lysine or L-arginine	starch	—	26.3	[112]
Ag	AgNO_3_	gum kondagogu	gum kondagogu	—	3	[54]
Ag	AgNO_3_	amino acid	amino acid	—	ND	[22]
Au	HAuCl_4_	POM^t^	POM^t^	—	10	[151]
Pd	K_2_PdCl_4_	POM^t^	POM^t^	—	15–50	[150]
Ag, Au, Pd and Pt	AgNO_3_, HAuCl_4_, PdCl_2_ and H_2_PtCl_6_	POM^t^	POM^t^	—	13 (Au), 15 (Ag), 5 (Pd) and 2.7-24 (Pt)	[134]
Au	HAuCl_4_	POM^t^	POM^t^	—	9.5	[36]
Pd and Pt	K_2_PtCl_4_, K_2_PdCl_4_, and PdSO_4_	POM^t^	POM^t^	—	1.7–4	[153]
Pd	[PdCl_4_]^2-^	POM^t^	POM^t^	—	3	[154]
Pd and Pt	K_2_PtCl_4_ and K_2_PdCl_4_	POM^t^	POM^t^	—	9-14 (Pd) and 1.7–3 (Pt)	[155]
Pt–Pd/GNs	K_2_PdCl_4_ and K_2_PtCl_4_	ethanol	—	GN^u^	7.9	[107]
Ag	AgNO_3_	geraniol	PEG^p^	—	1–10	[47]
Ag	AgNO_3_	NRL^u^	NRL^u^	—	2–100	[8]
Pd–Ag/RGO	AgNO_3_ and K_2_PdCl_4_	GO^v^	GO^v^	GO^v^	<10	[105]

^a^chitosan–ninhydrin: CHIT–NH; ^b^carboxymethyl chitosan: CMC; ^c^oligochitosan: (GlcN)*x*; ^d^sodium dodecyl sulfate: SDS, ^e^polyoxyethylenesorbitan monooleate: Tween 80; ^f^cetyltrimethylammonium chloride: CTAC; ^g^xylose, glucose, fructose and maltose; ^h^polyvinylpyrrolidon: PVP 360; ^i^degraded pueraria starch: DPS, ^j^hyaluronan acid: HA, ^k^2,6-diaminopyridinyl heparin: DAPHP; ^l^carboxymethyl cellulose sodium: CMS; ^m^diethylaminoethyl–dextran: DEAE–Dextran; ^n^calcium alginate: CA; ^o^sulfated polysaccharide: SP; ^p^polyethylene glycol: PEG; ^q^iron acetylacetonate: Fe(acac)_3_; ^r^tannic acid: TA; ^s^graphene: GN; ^t^polyoxometalates: POM; ^u^natural rubber latex: NRL, ^v^graphene oxide: GO.

### Phytochemicals

Phytochemicals are compounds that occur in plants and exhibit preventive or protective properties with regard to human health. They are not essential for the human body to survive but they can act as antioxidant, enzyme stimulator, or antibacterial agent, and they can interfere with DNA to prevent the multiplication of cancer cells. Researchers found that several phytochemicals, such as terpenoids and flavonoids can be used in the reduction of metal precursors to NPs [67,156]. This synthesis method has the advantages of other biological methods including low cost and being environmentally friendly [157]. However, they should be thoroughly studied for specific applications.

#### Plant-derived components

Leela and Vivekanandan investigated the capability of leaf extracts of different plants including *Helianthus annus*, *Basella alba*, *Oryza sativa*, *Saccharum officinarum*, *Sorghum bicolour* and *Zea mays* for the reduction of Ag precursor. They found that *H. annus* has strong potential for the reduction of Ag ions and is therefore promising in the development of Ag NPs [50]. Also Song and Kim used five plant leaf extracts including *Pinus desiflora*, *Diopyros kaki*, *Gingko biloba*, *Magnolia kobus* and *Platanus orientalis* for the synthesis of Ag NPs from AgNO_3_. They found that the extract of *Magnolia kobus* was the best reducing agent for synthesis of Ag NPs. They observed that for *Magnolia Kobus*, the final conversions were 60 and 100% at 25 and 55 °C, respectively, and the average particle size ranged from 15 to 500 nm [7].

Begum et al. investigated the performances of three different aqueous extracts of black tea in the formation of Ag and Au NPs from AgNO_3_ and HAuCl_4_. They used the extracts of compounds that were soluble in either water, ethyl acetate (C_4_H_8_O_2_), or dichloromethane (CH_2_Cl_2_) for the reduction of precursors and stabilization of NPs. They observed that the first two extracts can efficiently lead to a rapid formation of stable NPs with different shapes including spheres, trapezoids, prisms and rods. While, in the case of the third extract, no NP generation was detected under similar reaction conditions. Therefore, they concluded that polyphenols, such as flavonoids that are soluble in water and ethyl acetate, but are insoluble in dichloromethane are responsible for metallic ion reduction [24]. In another investigation, Moulton et al. used aqueous tea extract at different concentrations to reduce AgNO_3_ to Ag NPs at room temperature and obtained spherical NPs with controllable size (11 to 30 nm). According to their microscopy analysis, they suggested that keratinocytes are responsible for the stabilization of NPs [32]. Also, Nadagouda et al. used coffee and tea extracts to produce Ag and Pd NPs from AgNO_3_ and PdCl_2_ at room temperature. They obtained NPs in the size range of 20–60 nm and suggested that the Ag and Pd NPs were capped and stabilized by organic molecules such as polyphenols and caffeine [51]. In another study, Nune et al. used aqueous tea extract to reduce NaAuCl_4_ to Au NPs within 10 min. Their particles were spherical and in the size range of 15–45 nm [79].

Awwad and Salem worked on several phytochemicals with reducing capability to produce Ag NPs from AgNO_3_ at room temperature. They used aqueous extract of mulberry leaves in the reduction process of AgNO_3_ for 60 min. The produced NPs were spherical and ranged from 20 to 40 nm [58]. In another work, Awwad et al. used the aqueous extract of carob leaf (*Ceratonia siliqua*) as reducing and stabilizing agents in a 2 min reaction. The polydispersed NPs were spherical, and their sizes ranged from 5 to 40 nm with an average size of 18 nm. Their FTIR study showed that the carboxyl, hydroxyl, and amine groups in the both leaf extracts are accountable for the reduction of Ag^+^ ions to Ag NPs and the protein portion of leaf extract can play the role of both reducing agent and stabilizer for Ag NPs [45].

Ravindra et al. used aqueous extracts of *Eucalyptus citriodora* and *Ficus bengalensis* to produce Ag NPs with the size of around 20 nm at room temperature within 2–5 min. They conducted two different experiments under sunlight and in dark and observed that sunlight does not have any significant effect on the formation of Ag NPs [67]. In the same work, Saxena et al. employed the leaf extract of *Ficus benghalensis* as reducing and stabilizing agent and for production of Ag NPs in 5 min at 50–60 °C. Their analysis showed that phenolic compounds with hydroxyl and ketonic groups are responsible for reduction of Ag ions. The synthesized particles were monodispersed and spherical with a diameter range of 16 nm [13].

D. Philip employed an aqueous leaf extract of fresh/dry *Mangifera indica* as a reducing agent for the synthesis of nearly monodispersed spherical Au NPs from HAuCl_4_ under ambient conditions. The reaction time was 2 min and she obtained NPs with an average size of around 18 nm. It was found that the colloidal product was stable for more than five months. Moreover, D. Philip observed that dried leaf extract resulted in smaller and more uniformly distributed particles in comparison to fresh ones [158]. She also employed this extract for the synthesis of Ag NPs from AgNO_3_ at two different temperatures and pH values and found that an increase of pH and temperature accelerated the reaction and influenced the morphology of particles. According to the results, at pH 8, there are well-dispersed triangular, hexagonal and nearly spherical NPs with an average size of 20 nm. She also identified flavonoids, terpenoids and thiamine as the reducing compounds present in *Mangifera indica* [43]. In another work, she used the leaf extract of *Hibiscus Rosa sinensis* as a reducing agent for the synthesis of Ag and Au NPs. The ratio of metal salt to extract influenced the size and shape of Au NPs. She observed triangular, hexagonal, dodecahedral and spherical shapes for Au NPs. In case of Ag NPs, she found that changing the reaction medium pH in the range of 6.8 to 8.5 resulted in different shapes. The FTIR spectra revealed that Au NPs display interaction with amine groups and the Ag NPs with carboxylate ion groups [84].

Noruzi et al. used the aqueous extract of rose petals as reducing agent for production of HAuCl_4_ to Au NPs within 5 min at room temperature and investigated the effects of concentrations of Au precursor and extract. Their TEM micrographs and XRD patterns showed that the synthesized NPs had various shapes with average size of 10 nm. FTIR study showed that primary amine, carbonyl, hydroxy and other functional groups are involved in the reduction of the precursor and stabilization of NPs [20]. Nagajyothi et al. synthesized Ag and Au NPs from AgNO_3_ and HAuCl_4_ by using the aqueous extract of *Lonicera japonica* flower as a reducer and a stabilizer at 70 °C for 30–60 min. They obtained spherical, triangular and hexagonal Ag and Au NPs with average sizes of 7.8 and 8.02 nm, respectively [25].

Sulaiman et al. prepared the leaf extract of *Eucalyptus chapmaniana* (*E. chapmaniana*) to produce Ag NPs from AgNO_3_ at 50 °C for 60 min. The average sizes of produced NPs were estimated to be around 60 nm determined by using the Scherrer equation [6]. Smith et al. used leaf broth of *Cinnamomum zeylanicum* to reduce HAuCl_4_ to Au NPs in 60 min reaction. Within this reaction, a mixture of Au nano prisms and spheres were formed so that lower concentrations of the extract resulted in more prism–shaped particles, while higher concentrations the favored formation of spherical particles. The average particle size was around 25 nm at higher concentrations of the extract. According to the FTIR study, they concluded that enzyme or proteins of leaf broth can reduce the Au ions [39].

Gnanasangeetha and SaralaThambavani investigated the effect of aqueous leaf extract of *Corriandrum sativum* on the production of ZnO NPs from Zn(CH_3_COO)_2_ with NaOH at room temperature for 2 h. According to their results, using this phytochemical compound can stabilize the NPs and reduce the particle size from 81 to 66 nm [46]. Zhan et al. simultaneously reduced HAuCl_4_ and PdCl_2_ by aqueous leaf extract of *Cacumen Platycladi* to produce Au–Pd bimetallic NPs with an average size of 7 nm. The reaction took place for 2 h and the C=O and C–O groups in the extract stabilized NPs. They also concluded that the water-soluble polyhydroxy biomolecules, such as flavonoids and sugars, are accountable for the reduction of metallic ions [106]. Swamy et al. reduced AgNO_3_ to Ag NPs using methanolic leaf extract of *Leptadenia reticulata* (*L. reticulata*) at room temperature for 8 h. The produced NPs were spherical and their sizes ranged from 50 to 70 nm. They attributed the reduction of Ag ions to phenolics, terpenoids, polysaccharides, and flavone compounds [49]. Dipankar and Murugan synthesized Ag NPs from AgNO_3_ by utilizing the aqueous leaf extracts of *Iresine herbstii* as reducing agent. The process was carried out in dark and at room temperature but it took seven days to complete. The produced NPs were poly dispersed and their sizes ranged from 44 to 64 nm [48].

Shameli et al. extracted the tuber powder of *Curcuma longa* (*C. longa*) with water for reducing AgNO_3_ to Ag NPs at room temperature (25 °C) for 24 h. The produced NPs had an average diameter of 6.30 nm. From FTIR spectra, they concluded that the aldehyde groups in *C. longa* were involved in the reduction of Ag ions and other groups, such as hydroxyl (–OH), amine (–NH) and aliphatic C–H involved in the capping of the NPs [42]. In another study, they extracted the stem bark of *Callicarpa maingayi* into a methanol/water solution to use it as reducing and stabilizing agent. This time, Ag NPs were spherical with the average diameter of 12.40 nm and same functional groups were identified to be involved in reduction and stabilization processes [115]. Zargar et al. synthesized spherical Ag NPs with an average size of 18.2 nm using methanolic leaf extract of *Vitex negundo* (*V. negundo*) as a reducing agent for AgNO_3_ in a 48 h reaction at room temperature. Their results showed that *V. negundo* played an important role in the reduction and stabilization of Ag ions to Ag NPs [5]. In comparison to other investigations, it seems that the reaction rate of these two procedures at room temperature is not quite enough to implement in practical applications.

Kumar et al. studied the effect of pH on reduction of AuCl_3_ to Au NPs in the presence of aqueous leaf extract of *Cassia auriculata* within 10 min at room temperature (28 °C). They found that changing pH in the range of 3.4–10.2 had no effect on the stability of the Au NPs. The produced NPs in pH 3.4 were a mixture of triangular and spherical shape with sizes of 15–25 nm. [74]. Also Mata et al. investigated the effect of pH on the reduction performance of biomass of the brown algae *Fucus vesiculosus* in the solution of HAuCl_4_ at room temperature (23 °C). They found that maximum uptake was obtained at pH 7 and hydroxyl groups in the algal polysaccharides were accountable for Au reduction [113].

Singh et al. synthesized Ag NPs from AgNO_3_ using the aqueous leaf extract of *Argemone maxicana* as reducing and stabilizing agent at room temperature for 4 h. The XRD study showed that the produced Ag NPs has a mixture of cubic and hexagonal structures with the average size of 30 nm [9]. Das et al. used ethanolic leaf extract of *Centella asiatica* as reducing and stabilizing agent to synthesize Au NPs by reduction of HAuCl_4_ at room temperature (25 °C). TEM studies showed the particles to be of various shapes and sizes. They observed that Au NPs had an average size range of 9.3–10.9 nm and they were stabilized by a coating of phenolic compounds [88].

Bar et al. synthesized Ag NPs from AgNO_3_ by using the water dispersion of extract of *Jatropha curcas* as reducing and stabilizing agents. This reaction is completed in 15 min at 80 °C. They observed that the particles had diameter of 20–40 nm and were stabilized by the cyclic peptides present within the dispersion. FTIR showed peaks for carbonyl groups of the acid groups of different fatty acids, amide I and II which are responsible for reduction of Ag ions and stabilization of Ag NPs [76]. In another work, they carried out same experiment using aqueous seed extract of *Jatropha curcas* as reducing and stabilizing agents. They observed that by changing to AgNO_3_ particles with diameter ranging from 15 to 50 nm can be produced. Similarly, they identified same functional groups in *Jatropha curcas* for reduction of Ag ions [102].

Banerjee et al. used the leaf extracts of three different plants, *Musa balbisiana* (banana), *Azadirachta indica* (neem) and *Ocimum tenuiflorum* (black tulsi), to reduce AgNO_3_ to Ag NPs in a microwave oven over 4 min discontinuously. The smallest NPs were obtained with banana leaf extracts (80.2 nm). According to their FTIR study, compounds, such as flavonoids and terpenoids are responsible for stabilization of Ag NPs [109]. Basha et al. synthesized spherical Au NPs with a size of 4–24 nm using the extract of *Psidium guajava* (*P. guajava*). They used UV–vis spectrometry, FTIR, NMR and GC-MS to analyze the extract of *P. guajava* and found that guavanoic acid is the responsible compound for the reduction to Au NPs [75].

Jha et al. investigated three different plant extracts, *Bryophyllum sp.*, *Cyprus sp.* and *Hydrilla sp.*, to reduce AgNO_3_ to Ag NPs at 40 °C in a 10 min reaction. The produced NPs ranged from 2 to 5 nm. They concluded that the reduction of Ag ions were carried out by water soluble compounds, such as flavones, quinones and organic acids including oxalic, malic, tartaric and protocatechuic [159]. They also used *Eclipta* leaves to reduce the same Ag precursor and produced spherical particles in the range of 2–6 nm [160].

Krpetic et al. extracted two components from Cape aloe, namely aloin A and aloesin, to act as stabilizers in the synthesis of Au and Ag NPs from NaAuCl_4_ and AgNO_3_. They studied the effects of temperature, reaction time, and reducing agent concentration on particles size and shape of NPs. By changing the concentration of reducing agent (NaBH_4_) from 0.1 to 0.01 M, and temperature from 25 to 55 °C, the average size of Au NPs increased from 4 to 45 nm for aloesin from 6 to 35 nm for aloin A [161]. Wang et al. used the aqueous extract of *Scutellaria barbata* as the reducing agent for HAuCl_4_ and observed that 3 h is required for the conversion of most of Au ions to Au NPs in the size range of 5–30 nm at the room temperature [77]. Xie et al. used aqueous extract of algae *Chlorella vulgaris* for reduction of AgNO_3_ to Ag nanoplates in a 12 h reaction at room temperature. The thickness of the Ag nanoplates was 20 nm and the algal proteins were found to be responsible for the reduction of Ag ions to Ag nanoplates [59].

Chandran et al. used aqueous leaf extract of *Aloe vera* to reduce HAuCl_4_ to triangular Au NPs. They claimed that the employed procedure has control over the size of the triangular Au NPs in the range of 50 to 350 nm, by adjusting the concentration of the *Aloe vera* extract, which is favored for tuning their optical properties. Size of Au NPs can be controlled [41]. Also, Shankar et al. produced triangular Au NPs from HAuCl_4_ using the extract of the lemongrass plant as reducing agent and observed that the produced NPs have considerable absorption in the near-infrared (NIR) region [40]. They also used the proteins/enzymes extracted from leaves of Geranium (*Pelargonium graveolens*) to reduce Ag ions to Ag NPs with an average size of 27 nm [162]. Gardea-Torresdey et al. studied the reducing capability of alfalfa biomass for production of Au NPs from solutions of KAuCl_4_. The microscopic analysis showed five different types of Au NPs including FCC tetrahedral, hexagonal platelet, icosahedral multiple twinned, decahedral multiple twinned and irregular shaped particles. They also observed that smaller NPs were formed in low pH (ca. 2) [163]. Aromal and Philip fabricated Au NPs from HAuCl_4_ using the aqueous extract of fenugreek seeds (*Trigonella foenum-graecum*) as reducer and stabilizer. In their process, NPs with different sizes from 15 to 25 nm can be produced by adjusting the dominant parameters, such as pH and extract amount. The FTIR study showed that flavonoids are accountable for reduction of Au ions and proteins are involved in stabilization of NPs [19]. In a similar study, they used the extract of *Macrotyloma uniflorum* (*M. uniflorum*) as a reducing agent for production of Au NPs and studied the effects of extract concentration, temperature and pH on the formation of NPs. According to their results, the reduction rate is very high at 100 °C and the product is more stable at pH 6 in comparison to other conditions. The FTIR study showed that phenolic compounds involved in reduction and the proteins stabilized the NPs [14]. A summary of different plants used for NPs synthesis is presented in Table 3.

**Table 3:** Important examples of nanoparticle biosynthesis using plants.

plant	NP material	size (nm)	morphology	refs.

alfalfa	Au	up to 360	fcc^1^ tetrahedral, hexagonal platelet, icosahedral, decahedral and irregular	[163]
*Aloe Vera*	Au	50–350	spherical and triangular	[41]
aloin A and aloesin	Au	4–45	spherical	[161]
aloin A and aloesin	Ag	5	spherical	[161]
*Argemone maxicana*	Ag	30	cubic and hexagonal	[9]
*Azadirachta indica* (neem)	Ag	up to 200	triangular	[109]
black tea extracts	Ag and Au	≈20	spheres, trapezoids, prisms and rods	[24]
*Bryophyllum sp.*	Ag	2–5	fcc^1^ unit cell structure	[159]
*Cacumen platycladi*	Au–Pd	7	spherical	[106]
*Callicarpa maingayi*	Ag	12.4	spherical	[115]
*Cassia auriculata*	Au	15–25	triangular and spherical	[74]
*Centella asiatica*	Au	9.3–10.9	triangular, hexagonal and spherical	[88]
*Ceratonia silique*	Ag	5–40	spherical	[45]
*Chlorella vulgaris*	Ag	20	truncated triangular and irregular	[59]
*Cinnamomum zeylanicum*	Au	25	prisms and spheres	[39]
*Corriandrum sativum*	ZnO	66–81	cubic	[46]
*Curcuma longa*	Ag	6.3	spherical	[42]
*Cyprus sp.*	Ag	2–5	fcc^1^ unit cell structure	[159]
*Eclipta*	Ag	2–6	spherical	[160]
*Eucalyptus chapmaniana*	Ag	60	fcc^1^ unit cell structure	[6]
*Eucalyptus citriodora*	Ag	≈20	spherical	[67]
*Ficus bengalensis*	Ag	≈20	spherical	[67]
*Ficus benghalensis*	Ag	16	spherical	[13]
*Fucus vesiculosus*	Au	NR	spherical	[113]
*Hibiscus Rosa sinensis*	Au	≈14	triangular, hexagonal, dodecahedral and spherical	[84]
*Hibiscus Rosa sinensis*	Ag	≈13	spherical	[84]
*Hydrilla sp.*	Ag	2–5	fcc^1^ unit cell structure	[159]
*Iresine herbstii*	Ag	44–64	spherical	[48]
*Jatropha curcas* (latex)	Ag	20–40	fcc^1^ unit cell structure	[76]
*Jatropha curcas* (seed extract)	Ag	15–50	spherical	[102]
lemongrass plant	Au	≈25	triangular	[40]
*Leptadenia reticulate*	Ag	50–70	spherical	[49]
*Lonicera japonica*	Ag	7.8	spherical, triangular and hexagonal	[25]
*Lonicera japonica*	Au	8.02	spherical, triangular and hexagonal	[25]
*Macrotyloma uniflorum*	Au	14–17	spherical	[14]
*Mangifera indica*	Au	18	spherical	[158]
*Mangifera indica*	Ag	20	triangular, hexagonal and spherical	[43]
*Mulberry* leaves	Ag	20–40	spherical	[58]
*Musa balbisiana* (banana)	Ag	80.2	spherical	[109]
*Ocimum tenuiflorum* (tulsi)	Ag	up to 200	cuboidal	[109]
*Pelargonium graveolens*	Ag	27	spherical and ellipsoidal	[162]
*Psidium guajava*	Au	4–24	spherical	[75]
rose petals	Au	10	spherical, triangular and hexagonal	[20]
*Scutellaria barbata*	Au	5–30	spherical and triangular	[77]
tea extract	Ag	11–30	spherical	[32]
tea and coffee extract	Ag and Pd	20–60	spherical	[51]
tea extract	Au	15–45	spherical	[79]
*Trigonella foenum-graecum*	Au	15–25	spherical	[19]
*Vitex negundo*	Ag	18.2	spherical	[5]

^1^fcc: face-centered cubic.

#### Food-derived reagents

Rastogi and Arunachalam used the aqueous extract of garlic (*Allium sativum*) for the production of Ag NPs from [Ag(NH_3_)_2_]^+^ within 15 min. They performed their experiment under bright sunlight and claimed that it can act as catalyst. The produced NPs were polydispersed and spherical with an average size of 7.3 nm. They suggested that the proteins of garlic are involved in stabilization of Ag NPs [164]. Also, Ahamed et al. used garlic clove extract for synthesis of Ag NPs from AgNO_3_ at 50–60 °C within 30 min. Their Ag NPs were spherical with an average diameter of 12 nm [15]. Li et al. extracted *Capsicum annuum* L. for its use as reductant for synthesis of Ag NPs from AgNO_3_. They obtained spherical NPs with mean size of 10 nm. The FTIR study showed that the proteins, which contain amine groups, act as reducing agent in the production of Ag NPs. Also they found that with increasing reaction time, the sizes of the NPs increase [30]. Amin et al. prepared methanolic extract of *Solanum xanthocarpum berry* (*S. xanthocarpum berry*) to use as the reducing and stabilizing agents for the production of Ag NPs from AgNO_3_. The size and shape of Ag NPs can be controlled by selecting the proper values for reaction parameters including reaction time, temperature and the volume ratio of *S. xanthocarpum berry* to AgNO_3_ solution. They could produce monodispersed and spherical NPs with 10 nm in size at *S. xanthocarpum berry* to AgNO_3_ ratio of 2:1 within 25 min at 45 °C [69]. D. Philip investigated the capability of honey for reduction of HAuCl_4_ at room temperature and stabilizing the produced NPs. According to these results, anisotropic and spherical nanocrystals with the average size of 15 nm can be produced within 3 h. The FTIR study revealed that fructose acts as the reducing agent and honey proteins bind to the Au surface through the amino groups to stabilize the NPs [81]. In another study, the aqueous extract of *Volvariella volvacea* was prepared to act as reducing and stabilizing agent in the synthesis process of Au, Ag and Au–Ag NPs from HAuCl_4_ and AgNO_3_. The reaction time for Au and Ag were reported to be 2.5h and 6 h, respectively. Au NPs ranged from 20–150 nm in size and had different shapes while Ag NPs were spherical with average size of 15 nm. Au NPs are bound to proteins through free amino groups and Ag NPs through the carboxylate group of the residue of amino acids [31]. Jain et al. prepared the aqueous extract of papaya fruit for synthesis of polydispersed Ag NPs from AgNO_3_ at room temperature for 5 h. The produced NPs had hexagonal shape with the average particle size of 15 nm. FTIR analysis showed ethers and polyols groups which are considered to be responsible for the reduction of Ag ions [60]. Shukla et al. produce Au NPs by the reduction of NaAuCl_4_ with aqueous soybean extracts at 25 °C for 4 h. Their TEM analysis showed that the average size of the Soy–Au NPs were 15 nm. Akin to many researchers, they identified amino acids as the reducing groups in the formation of Au NPs [78]. Kumar et al. extracted the water soluble portion of *Terminalia chebula* (*T. chebula*) fruit and employed it for synthesis of several metals and metal oxide NPs. In first study, they produced Ag NPs from Ag_2_SO_4_ within 20 min. TEM studies showed anisotropic NPs with less than 100 nm in size. They found that the hydrolysable tannins such as di/trigalloylglucose can be hydrolyzed to gallic acid and glucose that consequently act as reducing agent. Furthermore, oxidized polyphenols are responsible for stabilizing the NPs [61]. In the second study, they reduced HAuCl_4_ to Au NPs using aqueous seed extract of *T. chebula*. The reaction time was 20 s and NPs were anisotropic with the size range of 6 to 60 nm. This time, they identified hydrolysable tannins as the responsible agent for reductions and stabilization [85]. In their third work, they used FeSO_4_ and PdCl_2_ as precursor of FeO and Pd NPs at a pH around 2. The reaction time for FeO and Pd formation were 5 and 40 min, respectively. The TEM study showed amorphous iron NPs with less than 80 nm in size and cubic Pd NPs with less than 100 nm in size. They concluded that phytochemicals/polyphenols are responsible for reducing and stabilizing processes [16]. Singh et al. used the aqueous extract of *Dillenia indica* (*D. indica*) for producing Ag NPs from AgNO_3_. The particles size of these Ag NPs ranges from 40 to 100 nm. This fruit is a potent source of ascorbic acid, α-tocopherol, β-carotene and phenolic components [52]. These components may be accountable for the reduction of Ag ions. However, the researchers did not study the reduction mechanism. Armendariz et al. investigated the binding trend of Au^3+^ ions to Oat (*Avena sativa*) biomass in a 60 min reaction at different pH from 2 to 6. They observed that at pH 3 (optimum condition) about 80% of Au ions were adsorbed to biomass and Au NPs with average size of 20 nm and different shapes such as tetrahedral, decahedral and hexagonal were produced. They also found that the NPs produced at pH 2 are larger than NPs produced in pH 3 and 4. According to their analysis, functional groups such as carboxyl, amino and sulfhydryl that are present in the cell walls of the inactivated tissues of the plant, can be accountable for reduction of Au ions [165]. Lu et al. used pomelo peel as a source for the production of carbon NPs in a hydrothermal process at 200 °C for 3 h. The obtained NPs ranged from 2 to 4 nm and the quantum yield was 6.9% [93].

### Energy saving processes

#### Energy transfer

Generally, there is some energy consumption in NPs synthesis either for obtaining the required temperature or for the direct reduction of metallic ions. Each synthesis route should be optimized in terms of energy consumption, reaction time and quality of NPs. In recent years, researchers have been working on new energy transfer techniques such as microwave, ultrasonic, gamma, ultraviolet (UV), and ion radiation to simultaneously reduce the reaction time and energy requirement and enhance the control on size and shape of NPs due to uniform heating of these techniques [10,27].

Sudeep and Kamat used thionine as a sensitizing dye for the photoinduced reduction of AgNO_3_ by visible light. They produced NPs in less than 60 min with 20 nm in size. They found that NPs were stabilized by thionine [28]. In another study, Dubas and Pimpan employed a low power ultraviolet (UV) irradiation source (8 W) as a reducing system to produce Ag NPs from AgNO_3_. They also used poly(methacrylic acid) (PMA) as reducing and stabilizing agent and the reaction was completed within 60 min at room temperature. The TEM images showed spherical NPs with the average particle size of 8 nm [166]. Also, Shameli et al. reduced AgNO_3_ to Ag NPs by UV irradiation and they used montmorillonite (MMT) and CTS as template and stabilizer, respectively. They investigated the effect of UV irradiation time and according to their results, the average size decreases from 10.97 nm to 3.16 nm by changing irradiation time from 3 h to 96 h [10]. Although they did not use any chemical reducer or heat treatment, no information was provided about energy consumption rate for this process. Bogle et al. used electron beam with fluences of 2 × 10^13^ to 3 × 10^15^ e·cm^−2^ at an energy of 6 MeV to reduce AgNO_3_ in water and poly(vinyl alcohol) (PVA). They found that the size of the Ag NPs could be tuned from 60 to 10 nm in PVA solution, and from 100 to 200 nm in aqueous solution by changing the electron fluence from 2 × 10^13^ to 3 × 10^15^ e·cm^−2^ [167]. Abid et al. employed direct laser irradiation of AgNO_3_ aqueous solution with an average energy of 12–14 mJ per pulse. They also used sodium dodecyl sulfate (SDS) to stabilize the particles. According to the proposed mechanism, the reaction starts with formation of radicals in the solution by multiphoton excitation and the growth of particles is terminated depending on the concentration of SDS. Therefore, increasing the SDS concentration can accelerate the termination process and consequently reduce the size of NPs. However, by changing the [SDS]/[AgNO_3_] ratio from 0.2 to 40, the average size will change in the range of 13 to 16 nm [27].

Bensebaa et al. produced two different NPs namely CuInS_2_ and CuInSe_2_ using microwave irradiation of aqueous solution for 30 min that increase the temperature to 90 °C. They employed mercaptoacetic acid (MAA) as stabilizing agent. Their TEM images showed particles with less than 5 nm in size. They claimed that low temperature and uniform heating with microwave are important parameters for production of high quality CuInS_2_ [139]. Although they did not use any harmful solvent or reducer, the stabilizing agent is toxic.

Darroudi et al. reduced AgNO_3_ to Ag NPs using ultrasonic waves at room temperature in the presence of gelatin that act as a stabilizer. They investigated the effects of Ag^+^ concentrations, ultrasonication time, and ultrasonic amplitude on the size of NPs. They observed that smaller particle size can be obtained with higher ultrasonic amplitude and shorter ultrasonication time. Spherical Ag NPs with an average size of 3.5 nm were produced by 45 min sonication with an amplitude of 50 [33].

Ramnani et al. employed ^60^Co gamma radiation as reducing agent for the production of Ag nanoclusters on SiO_2_ support in aqueous suspension containing isopropanol. According to their explanation, radical OH is produced as a result of water radiolysis and this radical can react with isopropanol to form isopropyl radicals. The new radical will reduce Ag ions to Ag nanoclusters. They observed that the nanoclusters ranged from 10 to 20 nm and were stable in the pH range of 2–9 [44]. In another study, Chen et al. produced Ag NPs by ^60^Co gamma radiation of AgNO_3_ solution in the presence of CTS as stabilizing agent and isopropanol as free radical scavenger. They obtained NPs with average diameters of 4–5 nm under a fixed radiation dose of 40.9 Gy/min [168].

#### Other approaches

Yang et al. produced ZnO_2_ NPs from natural ore containing hydrozincite (Zn_5_(CO_3_)_2_(OH)_6_) using H_2_O_2_ as reducer in ambient temperature and pressure. The obtained NPs were in the size range of 3.1-4.2 nm. Although their reducing agent is not a green reagent, using the ore can reduce the energy consumption and costs [169].

Wang et al. applied the ionic liquid 1-(3-aminopropyl)-3-methylimidazolium bromide (IL-NH_2_), to reduce aqueous HAuCl_4_ to Au NPs with average diameter of 1.7 nm. The reaction took place at room temperature and they observed that IL-NH_2_ was also involved in stabilizing Au NPs through a weak interaction between Au and N atoms [103]. Zhang et al. reduced [Ag(NH_3_)_2_]^+^ in ethanol to Ag NPs using triblock copolymer of poly(ethylene oxide)–poly(propylene oxide)–poly(ethylene oxide) to induce reduction under ambient light illumination. They observed that higher concentrations of Ag precursor result in the narrower size distribution (10–20 nm) in comparison to lower concentrations (5–30 nm) [170].

## Conclusion

Green Chemistry is aimed to ensure that scientists would consider the health of the whole planet as a design criterion for manufacturing of different products. NPs are among emerging products that can revolutionize the human life and, therefore, it is of great interest to produce them through green routes before proceeding to large scale production. In this paper, the recent investigations of different researchers on green synthesis of NPs are reviewed. To sum up, there are many green options to prevent from using harmful reagents such as reducers, stabilizers and solvents. Also there are new techniques for transferring of energy to reacting molecules, such as microwave and UV irradiation to decrease energy and time requirement as well as enhancing the control over particle size.

However, many of the proposed methods suffer from non-uniformity in shape and polydispersity in particle size. Thus a further study with the following research activities is required:

1. Investigating the performance of other environmentally friendly materials, e.g., other plant extracts and food-derived compounds for use as reagents for NPs production.

2. Optimizing the process parameters including temperature, pH, mixing speed, concentration of reactants to achieve the best results for size distribution and uniform shape. In the case of plant extracts, the purification of effective compounds can also be useful.

3. Finally, the repeatability, efficiency and scale-up capability of the selected methods should be evaluated.
